# The potential of tirofiban: recommendation for further research on preventing early neurological deterioration in acute ischemic stroke

**DOI:** 10.1097/MS9.0000000000002774

**Published:** 2025-01-09

**Authors:** Khaled A. Moghib, IZERE Salomon

**Affiliations:** a Faculty of Medicine, Cairo University, Cairo, Egypt; b Medical Research Group of Egypt, Negida Academy, Arlington, MA, USA; c University of Rwanda College of Medicine and Health Sciences, Kigali, Rwanda

*Dear Editor*,

The recent article by Zhao et al. (2024) on the administration of intravenous (IV) tirofiban in patients with acute ischemic stroke (AIS) demonstrated a high safety profile and significantly improved the prognosis of early neurological deterioration. I commend the authors for conducting this critical study, which adds to the growing body of evidence on the role of antiplatelet therapy in AIS patients. The decision to administer IV tirofiban according to the well-established PRISM-PLUS protocol (well-established protocol for administering intravenous tirofiban) is prudent, as previous studies have demonstrated the relative safety of this approach. The rationale for continuing the infusion for 72 h to cover the high-risk window for neurological deterioration is well justified. However, the study’s open-label design raises some methodological concerns, as a double-blinded medication administration would have been feasible and may have minimized potential bias. While the findings are promising, the authors acknowledge several limitations that warrant further investigation. Specifically, the generalization of results to patients with cardioembolic stroke and other global populations is unclear. Furthermore, the safety and efficacy of IV tirofiban in AIS patients with National Institutes of Health Stroke Scale scores outside the studied range of 4-20 remains to be determined. A search of recent randomized controlled trials revealed limited studies in this area, with fewer than 4 relevant results on PubMed. Future research should aim to address these gaps by expanding the study population to include a broader range of stroke subtypes and neurological deficits. Critical endpoints, such as functional outcomes (e.g., modified Rankin Scale scores) and the incidence of hemorrhagic complications, should be considered. Comparative studies between IV tirofiban and other antiplatelet or antithrombotic therapies would also help elucidate the relative benefits of this approach^[1]^.

## Data Availability

Not applicable.
